# Kinetics of SARS-CoV-2 IgM and IgG Antibodies 3 Months after COVID-19 Onset in Moroccan Patients

**DOI:** 10.4269/ajtmh.22-0448

**Published:** 2022-12-12

**Authors:** Najlaa Assaid, Soukaina Arich, Hicham Charoute, Khadija Akarid, Mohamed Anouar Sadat, Abderrahmane Maaroufi, Sayeh Ezzikouri, M’hammed Sarih

**Affiliations:** ^1^ Service de Parasitologie et des Maladies Vectorielles, Institut Pasteur du Maroc, Casablanca, Morocco;; ^2^ Biochemistry, Biotechnology and Immunophysiopathology Research Team, Health and Environment Laboratory, Aïn Chock Faculty of Sciences, University of Hassan II Casablanca, Casablanca, Morocco;; ^3^ Research Unit of Epidemiology, Biostatistics and Bioinformatics, Institut Pasteur du Maroc, Casablanca, Morocco;; ^4^ Virology Unit, Viral Hepatitis Laboratory, Institut Pasteur du Maroc, Casablanca, Morocco

## Abstract

Coronavirus disease (COVID-19) caused by severe acute respiratory syndrome coronavirus 2 (SARS-CoV-2) poses serious global public health problems. Characterization of the immune response, particularly antibodies to SARS-CoV-2, is important for establishing vaccine strategies. The purpose of this study was to evaluate longitudinally the kinetics of anti-SARS-CoV-2 antibodies against spike protein (S1) for up to 3 months in a cohort of 169 COVID-19 patients. We enrolled COVID-19 patients at two regional hospitals in Casablanca, Morocco, between March and September 2021. Blood samples were collected and N-specific IgM and S-specific IgG levels were measured by a commercial Euroimmun ELISA. IgM antibodies were assessed 2–5 (D00), 9–12 (D07), 17–20 (D15), and 32–37 (D30) days after symptom onset; IgG antibodies were assessed at these time points plus 60 (D60) and 90 (D90) days after symptom onset. We found that at 3 months after symptom onset, 79% of patients had detectable SARS-CoV-2-specific IgG antibodies, whereas their IgM seropositivity was 19% by 1 month after symptom onset. The IgM level decreased to 0.34 (interquartile range [IQR] 0.19–0.92) at 1 month after symptom onset, whereas the IgG level peaked at D30 (3.10; IQR 1.83–5.64) and remained almost stable at D90 (2.95; IQR 1.52–5.19). IgG levels were significantly higher in patients older than 50 years than in those younger than 50 at all follow-up time points (*P* < 0.05). Statistical analysis showed no significant difference in median anti-S1 antibody levels among infected patients based on gender or comorbidities. This study provides information on the longevity of anti-SARS-CoV-2 IgM and IgG antibodies in COVID-19 patients.

## INTRODUCTION

In late 2019, a new *sarbecovirus,* called SARS-CoV-2 (severe acute respiratory syndrome coronavirus 2), emerged in Wuhan, China, causing a global pandemic that is currently underway.^1^ This zoonotic virus causes coronavirus disease (COVID-19), which primarily affects the respiratory tract and in some cases progresses to atypical pneumonia that can be fatal.^2^ Understanding immune responses, particularly humoral responses, is important for characterizing the pathogenesis of coronavirus disease and for developing of effective vaccines against SARS-CoV-2. The duration and persistence of antibody responses against SARS-CoV-2 are now being widely studied and especially in light of new vaccine approvals against the virus.^3^^–^^6^ In symptomatic patients with COVID-19, anti-SARS-CoV-2 antibodies are produced and the antibody levels were different depending on the severity of disease^7^^–^^9^; however, studies have shown that asymptomatic patients also develop the anti-SARS-CoV-2 humoral response.^10^^,^^11^ Several studies have shown that most patients with COVID-19 produce IgM and IgG antibodies within 2 weeks of symptom onset.^12^^,^^13^

To date, studies of the immune response to SARS-CoV-2 after natural infection have demonstrated that convalescent COVID-19 patients continue to have IgG antibodies after several months from the onset of symptoms, although neutralization activity decreases.^3^^,^^14^^,^^15^ The antibody response induced after natural infection allows more than 90% of patients to recover naturally.^16^ Several investigations have shown a continuous decrease in antibody titers to SARS-CoV-2 observed 3 months after symptom onset.^17^^,^^18^ Other data have shown that antibody levels decline during the first 6 months after infection, particularly in immunocompromised individuals.^13^^,^^19^ Currently, with the ongoing pandemic worldwide and with the emergence of reinfection cases reported in several countries,^20^^–^^22^ the waning of natural infection-induced antibodies is a global concern, especially with the emergence of new SARS-CoV-2 variants (1). The emergence of these variants, which include Alpha (B.1.1.7), Beta (B.1.351), Gamma (P.1), Delta (B.1.617.2), and Omicron (B.1.1.529),^23^^,^^24^ has challenged the scientific community because they may influence retransmission, disease severity, diagnosis, and prevention of infection. Humoral immunity plays an important role in protection against reinfection,^25^ therefore follow-up studies of immune responses after natural infection and characterization of long-term antibody responses are of great importance to estimate the immune effects of vaccination and also to determine possible revaccinations.

In this study, between March 18 and June 8, 2021, we enrolled 169 patients with COVID-19 who were followed over a 3-month period after symptom onset by an antibody immunoassay targeting the SARS-CoV-2 spike protein. The primary objectives of the study were to assess the kinetics of antibody responses produced by natural infection with SARS-CoV-2 during the first 3 months after infection in COVID-19 patients with a wide range of clinical manifestations and to assess the effect of age, gender, and comorbidities on antibody kinetics.

## MATERIALS AND METHODS

### Study design and patient enrollment.

A longitudinal study was conducted between March 18, 2021, and June 8, 2021. A total of 169 patients with COVID-19 were recruited in the marquees of two hospitals in Casablanca (Moulay Youssef Regional Hospital and Mohamed Bouafi Hospital), and a 3-month follow-up after of symptom onset was implemented. These participants provided serum samples for antibody testing to check the presence and persistence of immunity to SARS-CoV-2.

Study participants were adults, male and female, unvaccinated against COVID-19 with a positive quantitative reverse transcription polymerase chain reaction (RT-qPCR) test, symptomatic (up to 5 days after symptom onset), and able to provide informed consent. All sociodemographic information, known comorbidities, disease information (COVID-19) (date of first symptoms, clinical signs) were collected on a paper questionnaire.

All participants in this study gave informed consent before participating. The study protocol complied with the Helsinki Declaration. The Ethics Committee of the Mohammed VI University of Health Sciences in Casablanca approved the study. Under the general rules on data protection, the contact details of the subjects were kept confidential, and after collecting the samples, the names were deleted and replaced by patient codes.

### Sample collection.

Five milliliters of venous blood was collected in dry tubes to isolate the serum. Samples were collected from individuals for whom informed consent was obtained at six time points: 2 to 5 days of symptom onset (D00), 9 to 12 days (D07), 17 to 20 days (D15), 32 to 37 days (D30), 62 to 67 days (D60), and 92 to 98 days (D90). Study participants were classified into two groups as follows: one group of patients who became infected with COVID19 but were not vaccinated during 3 months of follow-up (infected, unvaccinated patients [IUP]) and a second group of patients who were vaccinated after becoming infected and recovered (vaccinated postinfection patients [VAPI]).

Patients who received the SARS-CoV-2 vaccine were excluded from follow-up of the humoral response induced by natural infection, and antibody levels in both groups were analyzed at two time points: D60, 2 months after symptoms onset (i.e., 1 month after vaccination for VAPI); and D90, 3 months after symptoms onset (i.e., 2 months after vaccination for IVPA).

After centrifugation at 900 *g* for 10 minutes, serum samples were separated and stored at –20°C for serological testing: IgM and IgG.

### Detection of IgG and IgM against SARS-CoV-2.

ELISA was used to detect IgM and IgG antibodies against the SARS-CoV-2 in human serum. We used the commercial Anti-SARS-CoV-2 S1 ELISA IgG kit and nucleocapsid protein-specific IgM kit (all by Euroimmun, Lübeck, Germany) following the manufacturer’s instructions.^26^^,^^27^ The ELISA IgG kit detects the S1 domain of the SARS-CoV-2 spike protein with a specificity of 99% and a sensitivity of 93.8%,^28^ whereas the IgM kit allows the detection of the nucleocapsid protein with a specificity of 98.6% and a sensitivity of 88.2%. Microplate wells are coated with recombinant S1 structural protein for IgG detection or nucleocapsid protein for IgM detection and the test detects anti-SARS-CoV-2 against viral proteins. Results were analyzed and interpreted according to the manufacturer’s instructions. The extinction ratio of the control or patient sample to the calibrator extinction. This ratio is interpreted as follows: < 0.8 negative; ≥ 0.8 to < 1.0 borderline; 1.1 positive. Borderline results were considered negative for analysis.

### Statistical analysis.

Categorical variables are presented as number and proportion. For descriptive statistics, continuous variables are shown as median and interquartile range (IQR). Comparisons of continuous variables between groups were conducted using Wilcoxon and Mann Whitney *U* tests. All statistical analyses were performed with the R software package (https://www.r-project.org). Values below 0.05 were considered statistically significant. All statistical tests were two-sided.

## RESULTS

### Clinical and demographic characteristics of enrolled patients at baseline.

A total of 169 participants infected with SARS-CoV-2, whose infection was confirmed by a positive RT-PCR test, were collected in our study. The demographic and clinical data of these patients are presented in Table 1. The median age of participants was 47 years (range: 30–57 years), and females predominated (106 [62.72%] versus 63 [37.28%] males). The majority of participants were adults under age 50 years (110 [65.09%]). The most common symptoms among the study participants were muscle pain (136 patients, 80.47%) followed by cough (122 patients, 72.19%), headache (116 patients, 68.64%), and chills (103 patients, 60/94%). Other symptoms included fever (52.07%), loss of appetite (44.37%), and sore throat (42.60%). Of the total of patients, 39 (23.07%) had preexisting diseases, and diabetes was the most common comorbidity (22 patients, 56.41%) (Table 1).

**Table 1:** Clinical and demographic characteristics of the study cohort (*N* = 169)

Characteristics	*n* (%)
Total	169 (100)
Gender	
Female	106 (62.72)
Male	63 (37.28)
Age, median (interquartile range), years	47 (30–57)
Age (years)	
18–35	66 (39.06)
35–50	44 (26.03)
> 50	59 (34.91)
Symptoms	
Fever	88 (52.07)
Cough	122 (72.19)
Sore throat	72 (42.60)
Chill	103 (60.94)
Headache	116 (68.64)
Asthenia	68 (40.23)
Nausea	48 (28.40)
Vomiting	21 (12.42)
Diarrhea	56 (33.13)
Abdominal pain	37 (21.89)
Loss of appetite	75 (44.37)
Muscle pain	136 (80.47)
Dyspnea	43 (25.44)
Comorbidities	39 (23.07)
Obesity	4 (10.25)
Diabetes	22 (56.41)
Asthma	8 (20.51)
Cardiovascular disease	5 (12.82)

### Kinetics of IgM and IgG antibodies.

**Figure 1.**
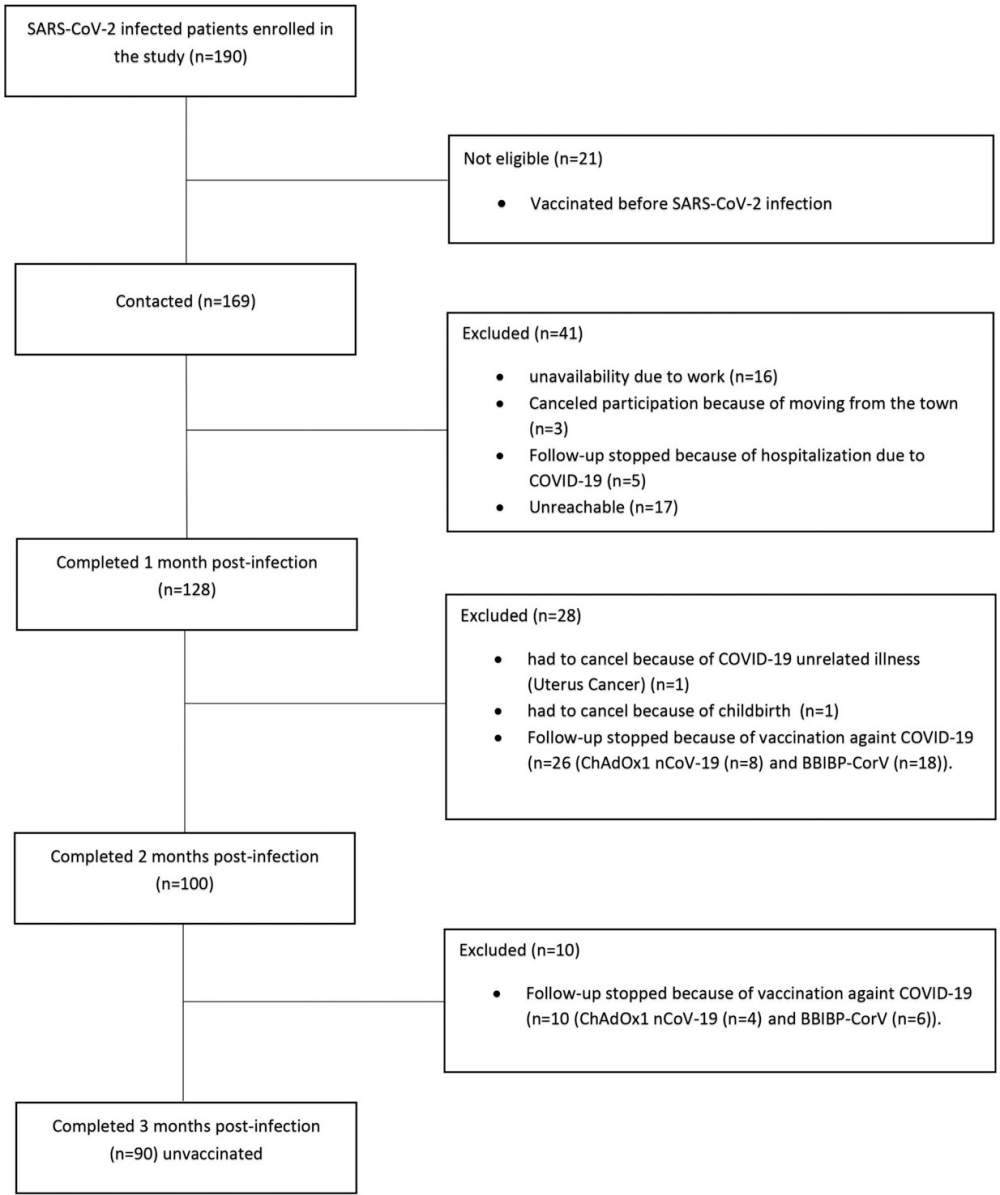
Study flowchart. BBIBP-CorV = vaccine Sinopharm; ChAdOx1 nCoV-19 = vaccine AstraZeneca; COVID-19 = coronavirus disease 2019; SARS-CoV-2 = severe acute respiratory syndrome coronavirus 2.

One hundred and sixty-nine patients diagnosed with SARS-CoV-2 infection by RT-PCR were enrolled in the present study. Only 90 patients, or 53.26% (90/169), completed the 3 months. Seventy-nine patients were excluded from the study for various reasons: moving from town, hospitalization, unavailability due to work or vaccination (Figure 1).

### IgM responses to SARS-CoV-2 during 1-month follow-up.

**Figure 2.**
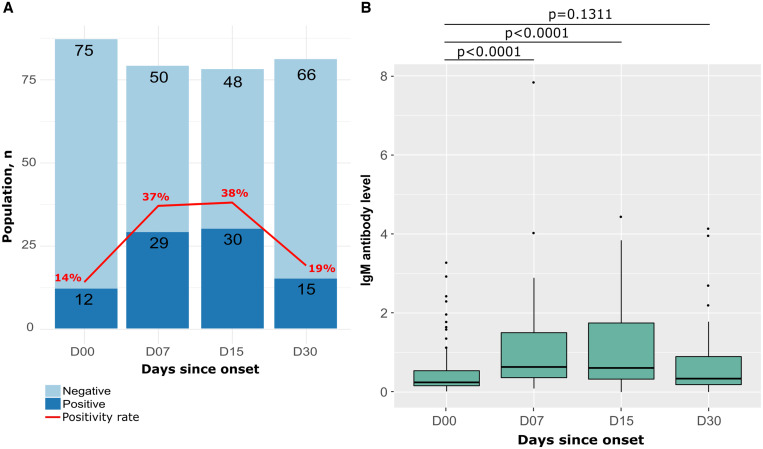
Seropositivity rates and median IgM antibody levels to SARS-CoV-2 as a function of days after symptom onset at follow-up. (**A**) Dark blue represents the number of IgM-positive participants; light blue represents negative participants. The red curve indicates the IgM seropositivity rate. (**B**) Boxplots depict the distributions and differences of anti-SARS-CoV-2 IgM antibodies obtained by Euroimmun ELISA for participants sampled at different follow-up periods. *P* values are calculated by the Wilcoxon and Mann–Whitney tests. SARS-CoV-2 = severe acute respiratory syndrome coronavirus 2.

IgM antibodies were measured in sera of COVID-19 patients for 1 month after onset of symptoms. The follow-up period was divided into four-time intervals: D00 (2–5 days after symptoms onset), D07, D15, and D30. Within 20 days of symptom onset (between D00 and D15), the IgM seropositivity rate fluctuated between 14% and 38%, and then this IgM positivity rate decreased to 19% at 1 month after symptom onset (Figure 2A). At the same time, the median IgM antibody level was initially 0.25 (IQR 0.16–0.58) and increased 1 to 2 weeks after symptoms onset to 0.69 (IQR 0.41–1.70), and then the IgM level decreased to 0.34 (IQR 0.19–0.92) 1 month after symptom onset. The differences in median antibody levels between D00 and D07 and between D00 and D15 were statistically significant (*P* < 0.0001). The median antibody level of antibodies decreased to almost the same value as at baseline 1 month after symptom onset (*P* = 0.131) (Figure 2B).

**Figure 3.**
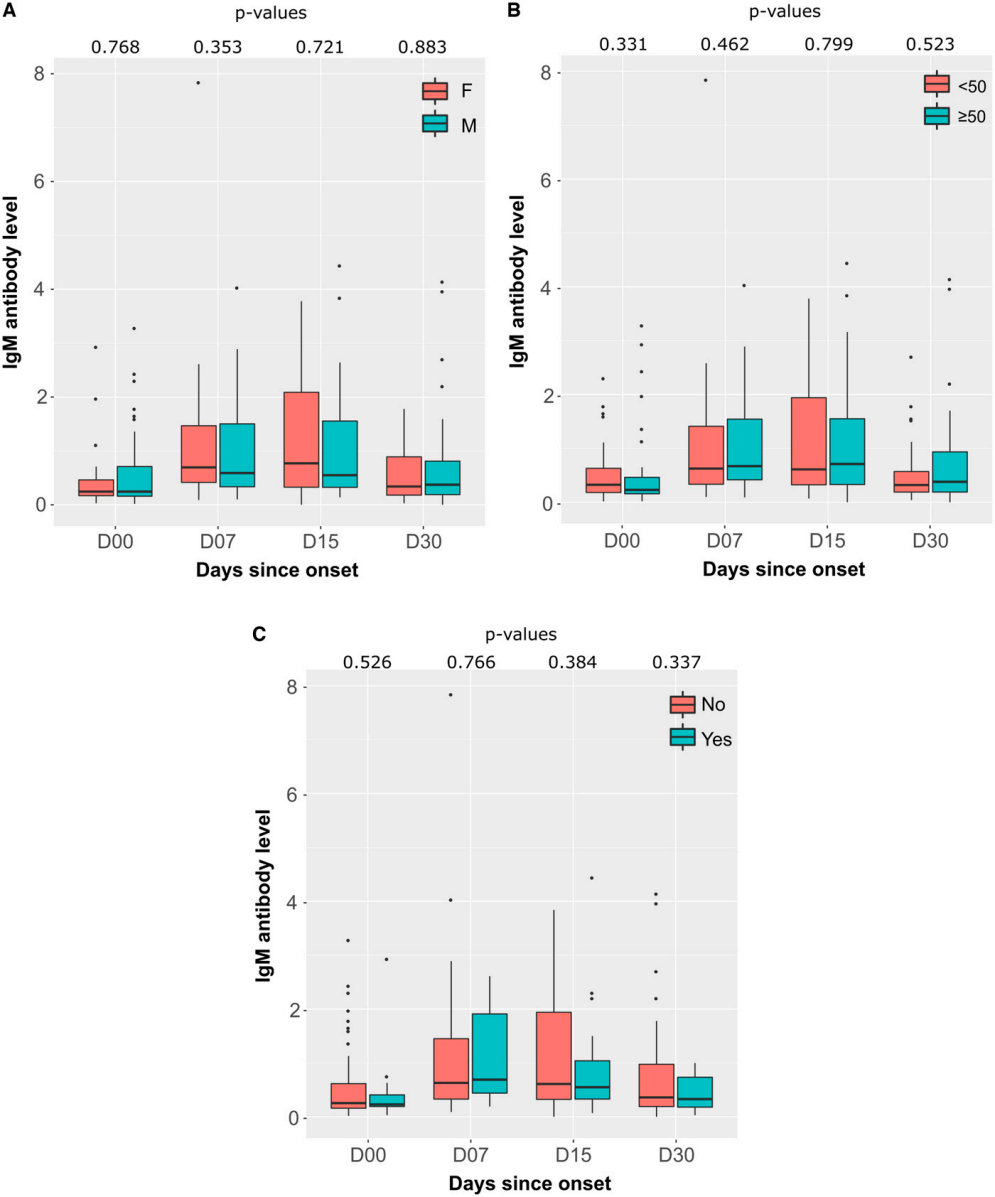
Distributions and differences of IgM antibodies to SARS-CoV-2 according to gender, age, and comorbidity at follow-up, obtained by the Euroimmun ELISA assay. The bold line is the median antibody value. (**A**) Comparison of SARS-CoV-2 IgM antibody level by gender (F = female; M = Male). (**B**) The median age of the 169 participants was 47 years (interquartile range: 30–57 years); patients were divided into two groups as follows: < 50 and > 50, shown with each age interval in the boxplots. (**C**) IgM antibody level presented with the presence or absence of comorbidities in the boxplots. *P* values are calculated by the Wilcoxon test. SARS-CoV-2 = severe acute respiratory syndrome coronavirus 2.

IgM response was assessed according to gender, age, and comorbidities (Figure 2). The median IgM antibody level was higher in female than in male at D15 after symptom onset (0.77 [IQR 0.33–2.09] versus 0.58 [IQR 0.33–1.69], respectively), but the difference was not statistically significant (*P* = 0.721). However, median rates at baseline and 1 month after symptom onset were similar between female and male (at D00: 0.26 [IQR 0.17–0.47] versus 0.25 [IQR 0.16–0.71]; at D30: 0.34 [IQR 0.19–0.91] versus 0.37 [IQR 0.19–0.81], respectively) (Figure 3A). The age range of the participants was 18 to 67 years and was divided into two groups: group 1 (< 50) and group 2 (> 50). Indeed, there was no difference in median IgM antibody level by age of participants except at 2 weeks after symptoms onset (D15), when there was a slight increase in median antibody level in those over 50 years of age compared with those under 50 years old with a statistically nonsignificant difference (0.77 [IQR 0.33–1.69] versus 0.61 [IQR 0.33–2.09], respectively; *P* = 0.799) (Figure 3B). Patients were classified according to the presence or absence of preexisting diseases. Patients with comorbidities had lower median antibody levels than those without comorbidities at 2 weeks after symptom onset (0.55 [IQR 0.33–1.04] versus 0.71 [IQR 0.34–2.08], respectively; *P* = 0.384), whereas the median antibody level was nearly similar in both patient groups at the other follow-up time points (*P* > 0.05) (Figure 3C).

### IgG responses to SARS-CoV-2 during 3-month follow-up.

**Figure 4.**
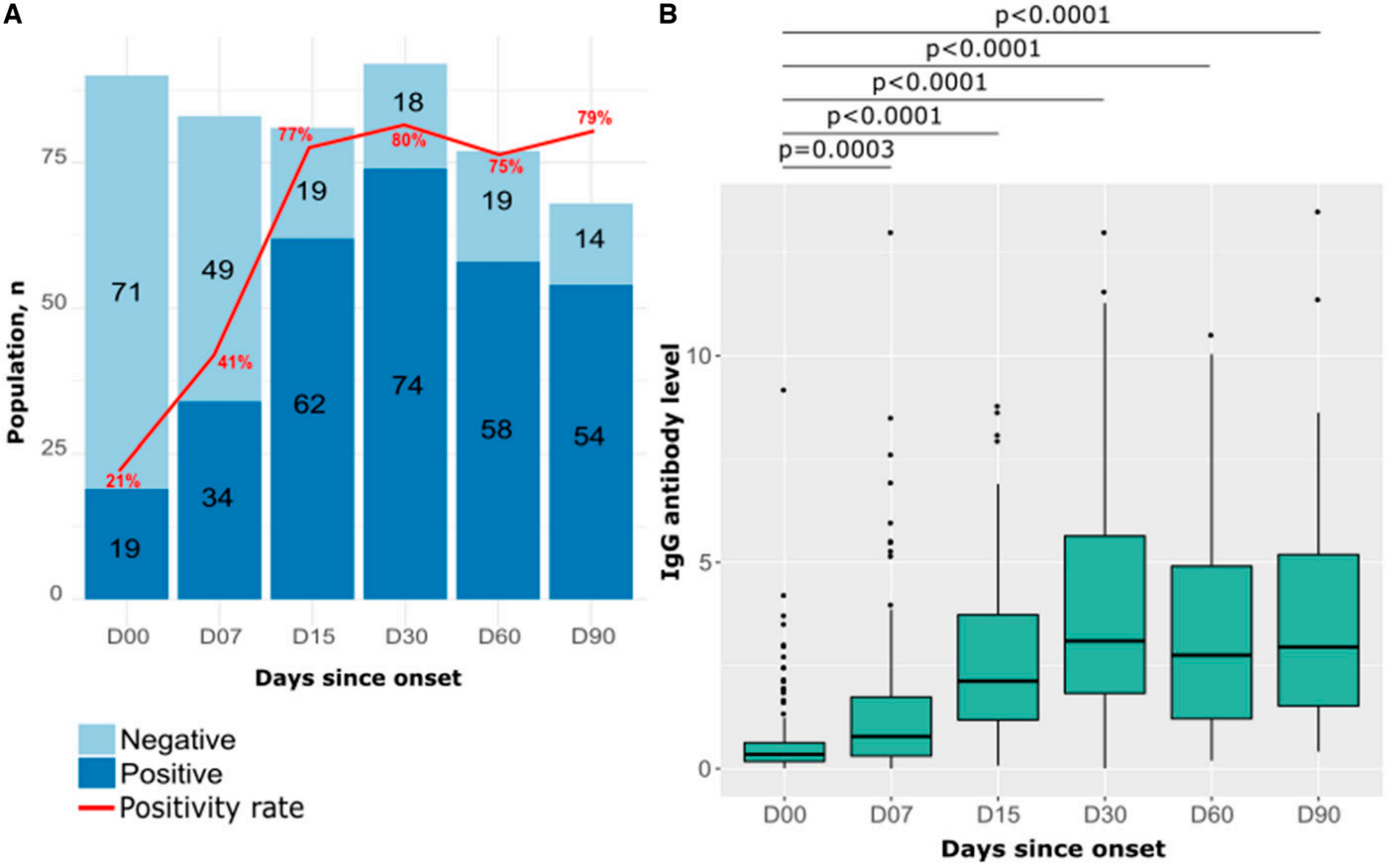
Seropositivity rates and median IgG antibody levels to SARS-CoV-2 by days after symptom onset at follow-up. (**A**) Dark blue represents the number of IgG-positive participants; light blue represents negative participants. The red curve indicates the IgG seropositivity rate. (**B**) Boxplots depict the distributions and differences of anti-SARS-CoV-2 IgG antibodies obtained by Euroimmun ELISA for participants sampled at different follow-up periods. *P* values are calculated by Wilcoxon and Mann–Whitney tests. SARS-CoV-2 = severe acute respiratory syndrome coronavirus 2.

The IgG antibodies level was analyzed at six time points after symptoms onset (2–5 days after symptom onset through 3 months of follow-up—D00, D07, D15, D30, D60, and D90). IgG antibody seropositivity to SARS-CoV-2 gradually increased over the follow-up period, from 21% at D00 to peak at 1 month after symptoms onset (80%). Between 1 and 3 months of follow-up, no decreasing trend was observed and the positivity rate fluctuated ∼77% to 80% (Figure 4A).

Median IgG levels in the D30- to D90-day intervals were significantly higher than those at D00 to D15 days (*P* < 0.0001) (Figure 3B). At baseline, the median IgG antibody level was 0.35 [IQR 0.17–0.63] and began to increase from 2 weeks after symptom onset (2.12 [IQR 1.18–3.73]), reaching its maximum level at D30 (3.10 [IQR 1.83–5.64]). IgG antibodies persisted 3 months after the onset of symptoms, with a median level slightly lower that noted at D30 (at D90: 2.95 [IQR 1.52–5.19]) (Figure 4B).

**Figure 5.**
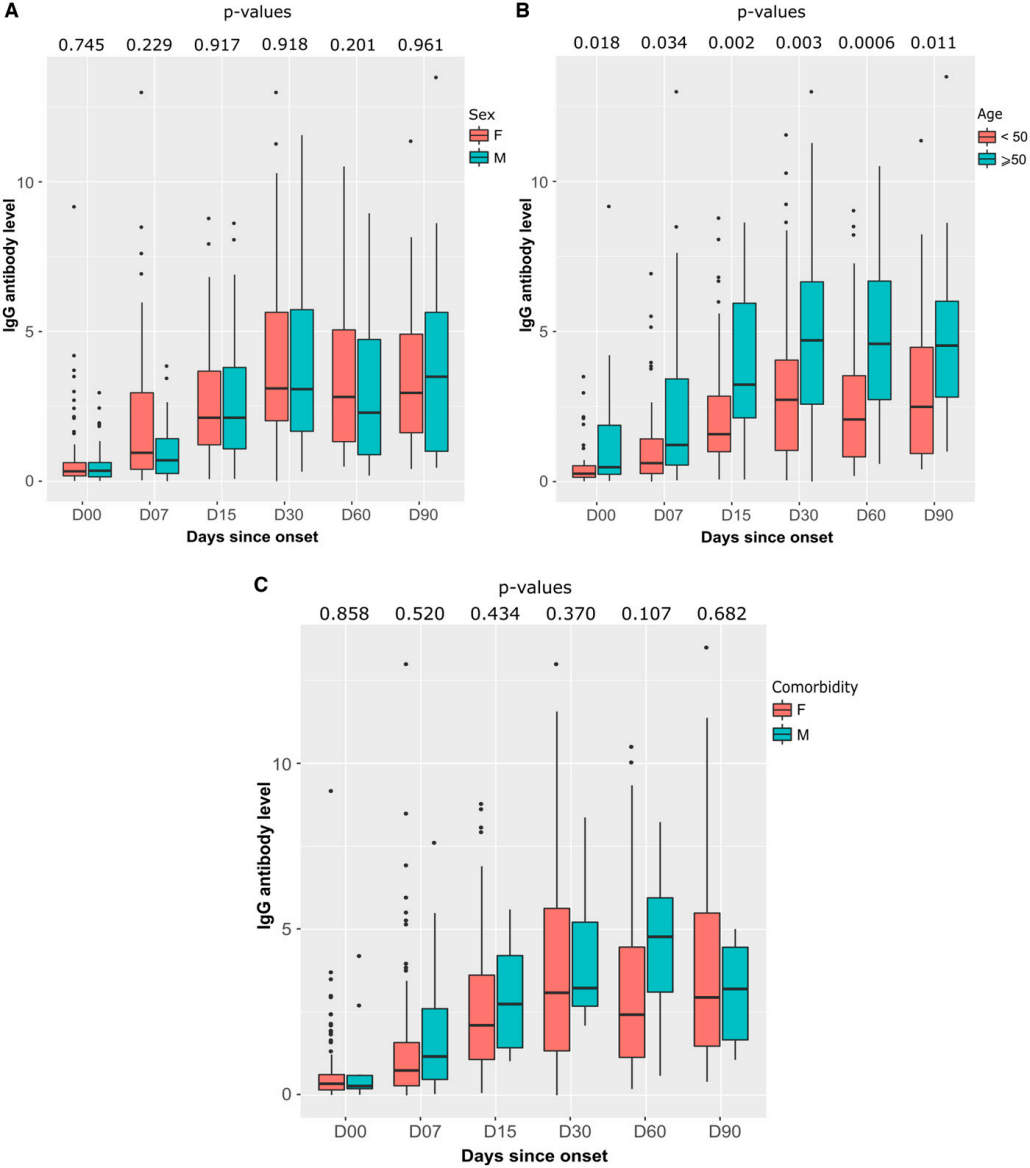
Distributions and differences of IgG antibodies to SARS-CoV-2 by sex, age, and comorbidity at follow-up, obtained by Euroimmun ELISA assay. The bold line is the median antibody value. (**A**) SARS-CoV-2 IgG antibody levels according to sex. (**B**) The median age of the 169 participants was 47 years (range: 30–57 years) and divided into two groups as follows: < 50 and > 50, shown with each age range in the boxplots. (**C**) IgG antibody level presented with the presence or absence of comorbidities in the boxplots. *P* values are calculated by the Wilcoxon test. SARS-CoV-2 = severe acute respiratory syndrome coronavirus 2.

Median IgG antibody levels were almost similar between female and male at different follow-up times. However, the median antibody level was higher in female than in male 2 months after symptom onset (2.82 [IQR 1.32–5.06] versus 2.29 [IQR 0.89–4.74], respectively; *P* = 0.201). At D90, the antibody level of male became higher than that of female (3.49 [IQR 1.00–5.64] versus 2.95 [IQR 1.62–4.91], respectively; *P* = 0.961) (Figure 5A). Patients older than 50 years had a very high median IgG antibody level compared with those younger than 50 years at all follow-up time points (*P* < 0.05) (Figure 5B). Participants with comorbidities had higher median IgG antibody levels than those without preexisting conditions, but the differences were not statistically significant (Figure 5C).

### IgG antibody level at two follow-up time points in two patient groups: IUP and VAPI.

**Figure 6.**
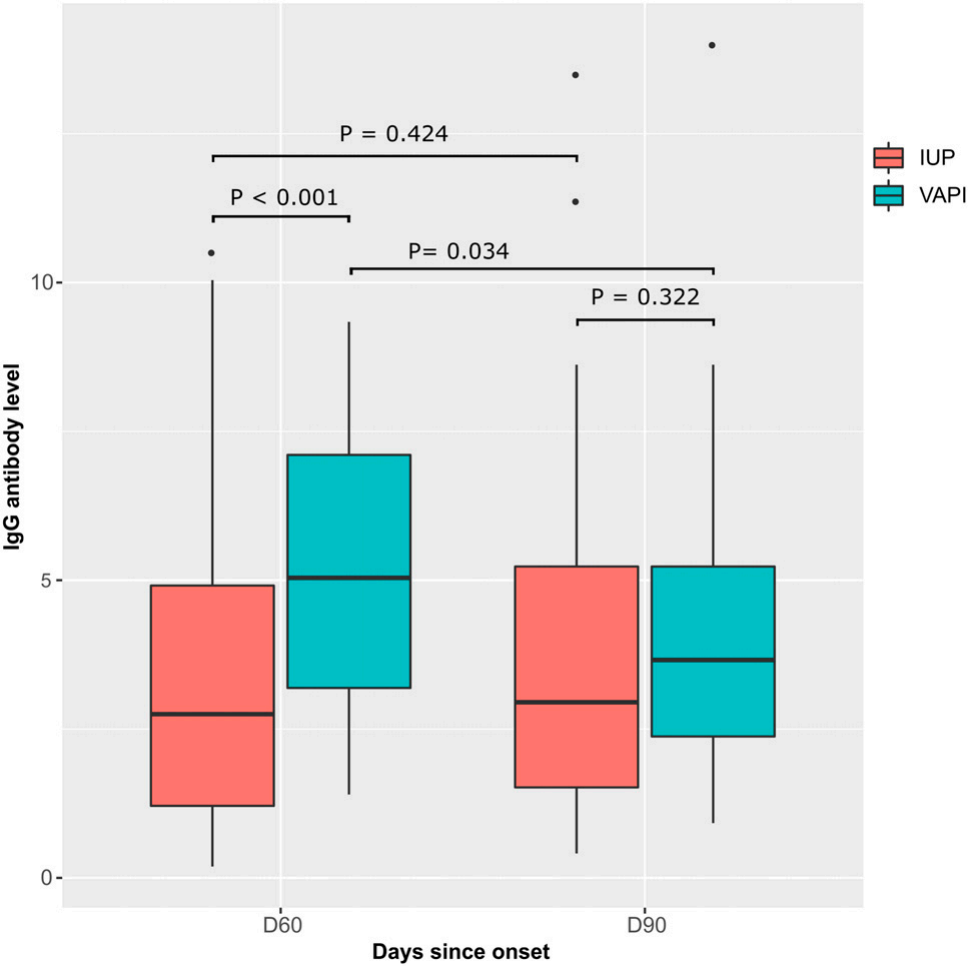
Comparison of IgG antibody levels between two groups of patients at two follow-up time points (D60 and D90). IUP = infected, unvaccinated patients; VAPI = vaccinated after infection patients. *P* values are calculated by the Wilcoxon test.

The antibody level was then analyzed in two groups of patients: IUP and VAPI. Patients in this second group (*n* = 36) received the vaccine 1 month after infection (ChAdOx1 nCoV-19; *n* = 12) or BBIBP-CorV (*n* = 24)). Antibody levels in both groups were analyzed at two time points: D60 (2 months after symptoms onset, i.e., 1 month after vaccination for VAPI) D90 (3 months after symptoms onset, i.e., 2 months after vaccination for VAPI) (Figure 6). At D60, the median IgG antibody level to SARS-CoV-2 was significantly higher VAPI than in IUP (5.040 [3.190–7.105] versus 2.750 [1.210–4.910], respectively; *P* < 0.001). At D90, this median antibody level remained high in VAPI compared with IUP, but the difference was not statistically significant (*P* = 0.322). No change was observed in the median antibody level in IUP at D60 and D90 (2.750 [1.210–4.910] versus 2.950 [1.520–5.230], respectively; *P* = 0.424)). In contrast, in VAPI, the antibody level decreased significantly at D90 compared with D60 (3.660 [2.375–5.230] versus 5.040 [3.190–7.105], respectively; *P* = 0.034)) (Figure 6).

## DISCUSSION

The interaction between the human immune system and SARS-CoV-2 induces the production of antibodies essential for sustained defense. Understanding this interaction is important not only for the prognosis of COVID-19 but also to improve infection control strategies, especially as the virus continues to mutate and generate multiple variants. These mutations increase virulence and transmissibility or allow the virus to evade the immune response.^29^ At an early stage of the disease, IgM antibodies are produced and respond rapidly establishing a short-term response; later, IgG is produced and prolongs the immune response, whereas the efficacy of the antibody response is determined by neutralizing antibodies. In the present study, we measured and monitored IgM the levels against SARS-CoV-2 in patients with COVID-19 at 1 month after symptom onset and IgG levels at 3 months after symptom onset to assess the kinetics of antibody responses produced by natural infection at several time points, the acute phases (1 week and 2 weeks after symptom onset), convalescent (1 month after symptoms onset), and post-convalescent (2 months and 3 months after symptoms onset) phases of the disease and to analyze the effect of age, gender, and comorbidities on antibody evolution over time.

Our data show that IgG antibody levels peak between 2 weeks and 1 month after symptom onset and then persist up to 90 days after symptom onset, whereas IgM levels tend to decline 1 month after SARS-CoV-2 infection. We also noted that antibody levels increased rapidly during the first 2 weeks after symptom onset. Previous studies have reported that seroconversion occurs within 2 weeks of symptom onset, similar to other viral infections.^13^^,^^30^ We found that at 3 months after symptom onset, 79% of patients had detectable SARS-CoV-2-specific IgG antibodies, whereas their IgM seropositivity was 19% by 1 month after symptom onset. A study of 308 healthcare workers who tested RT-PCR positive for COVID-19 showed that 99% of patients had anti-S antibodies 3 months after infection.^31^ In the present study, the median IgM level decreased to 0.34 [IQR 0.19–0.92] at 1 month after symptom onset, whereas IgG levels peaked at D30 (3.10 [IQR 1.83–5.64]), then persisted and remained stable at D90 (2.95 [IQR 1.52–5.19]). Some studies support our findings on IgG persistence, with one study of 343 patients showing that IgG persisted in patients for up to 3 months after symptom onset and IgM disappeared after 49 days.^32^ Another study by Isho et al. also reported that IgG remained relatively stable for up to 105 days after symptom onset, whereas IgM remained in serum for a short time.^33^ One study showed that IgM antibodies reached their peak levels at 1 month after symptom onset and then declined at 2 months after symptom onset.^5^ However, other data report persistence of antibodies to SARS-CoV-2 within 4 months of diagnosis.^34^ On the other hand, another study reports that SARS-CoV-2 specific antibodies rapidly degrade between 30 and 90 days after symptom onset.^5^^,^^35^ The observed discrepancy in results may be explained by the size of the cohort and the types of serological tests used for each study, including the sensitivity and specificity of each assay and the patient population studied, including the different clinical manifestations of COVID-19. SARS-CoV, which shares the same receptor and approximately 79.6% genomic sequence identity with SARS-CoV-2,^36^ has been shown to induce IgG levels that remain elevated for up 100 days after symptom onset,^37^ which is consistent with our current findings with SARS-CoV-2. The sustained presence of IgG for more than 2 years in patients cured of SARS-CoV has been described by some investigators,^38^^,^^39^ indicating the importance of monitoring the evolution of anti-SARS-CoV-2 antibodies not only to monitor their persistence over time. The study of antibody kinetics has also allowed the definition of booster vaccination, which is linked and determined according to epidemiological scenarios, including variants of concern. According to the WHO, in the short term, a third dose (booster dose) could fully or partially restore vaccine effectiveness of vaccines.^40^ It should be noted that factors such as gender, age, and comorbidities may have an impact on the antiviral humoral response and its persistence over time. In our study, statistical analysis showed no significant difference in median anti-S1 antibody levels in infected patients based on gender or comorbidity. In contrast to our results, gender differences in antibody response to SARS-CoV-2 have been described by several authors,^31^^,^^41^^–^^43^ some of whom have shown that male have higher antibody levels compared with female, but that these decline more rapidly soon after infection.^31^^,^^41^ Although we did not detect a difference between gender and antibody production, The size of our cohort does not allow us to conclude with great confidence that there is no difference in the level of antibodies between women and men. Thus we assume that the realization of other studies including larger samples will allow to better understand the association between the level of antibodies and the sex of the patient. Several studies show that female develop stronger immune responses against viruses and vaccines. This may be related to sex hormones, genetic factors (X chromosome), or environmental factors.^44^

In our study, IgG levels were significantly higher in individuals older than 50 years compared with other study participants during all phases of infection. Consistent with our findings, advanced age has been correlated with higher convalescent antibody titers in several studies.^25^^,^^42^^,^^45^^,^^46^ An increased viral load and an uncontrolled inflammatory state in the elderly could be the cause of more potent antibody production against SARS-CoV-2 than in younger subject. Despite all these theoretical concepts, the association between age, gender, and antibody responses requires additional and extensive analyses in a large cohort to be explore. In our study, we analyzed the antibody response in patients who received the SARS-CoV-2 vaccine and were excluded from the natural infection-induced humoral response follow-up (IUP) to compare with the group of patients who completed follow-up and did not receive vaccine (VAPI). We found the median level of antibodies was significantly higher in VAPI than in IUP but declined rapidly and remarkably in VAPI. The difference in median antibody levels between the two groups appears evident because in the VAPI group, the immune response induced by natural infection is enhanced by vaccination. The rapid decline in antibody levels in VAPI compared with IUP may be explained by the fact that antibodies induced after vaccination likely degrade more rapidly over time and have a shorter lifespan than those induced by natural infection. Previous infection with COVID-19 has previously been shown to cause robust and high levels of anti-SARS-CoV-2 antibodies in vaccinated individuals compared with unvaccinated people, but these antibodies decline rapidly over time.^47^^–^^49^ Vaccination-induced antibodies may not generate sufficient immune memory; therefore, repeated booster vaccinations at regular intervals appear necessary. Further studies are needed to understand the dynamics of immune response decline to vaccines and infections.

Our study has some limitation. First, given our limited resources, we did not have the opportunity to assess memory B- and T-cell responses, nor neutralizing antibody levels, making it difficult to correlate our results with protective antibody response or to have information on immune memory. Second, the follow-up period was short (only 3 months after symptom onset), although the originally defined follow-up period was 12 months; unfortunately, the recruited patients received their vaccines after the 3-month collection, and we had to stop the follow-up. Despite these limitations, this study is the first in North Africa to assess the durability of humoral immune responses against SARS-CoV-2 induced after natural infection.

In conclusion, our data show that anti-SARS-CoV-2 antibody responses persist for at least 3 months after symptom onset, and that IgG levels remain relatively stable during the post-convalescence period in COVID-19 patients. To establish a link between the presence of antibodies and the level of protection against SARS-CoV-2 reinfection, the kinetics of humoral and cellular anti-SARS-CoV-2 immunity need to be better characterized. These data should help health authorities to optimize vaccination strategies to define the timing of booster doses.
